# Atypical manifestation of secondary syphilis in a pediatric patient

**DOI:** 10.1590/0037-8682-0194-2023

**Published:** 2023-07-24

**Authors:** Gabriela Campos de Almeida, Simone Saintive, Gabriel Castro Tavares

**Affiliations:** 1 Universidade Federal do Rio de Janeiro, Hospital Universitário Clementino Fraga Filho, Serviço de Dermatologia, Rio de Janeiro, RJ, Brasil. Universidade Federal do Rio de Janeiro Hospital Universitário Clementino Fraga Filho Serviço de Dermatologia Rio de Janeiro RJ Brasil; 2 Universidade Federal do Rio de Janeiro, Instituto de Puericultura e Pediatria Martagão Gesteira, Serviço de Dermatologia, Rio de Janeiro, RJ, Brasil. Universidade Federal do Rio de Janeiro Instituto de Puericultura e Pediatria Martagão Gesteira Serviço de Dermatologia Rio de Janeiro RJ Brasil

**FIGURE 1:**
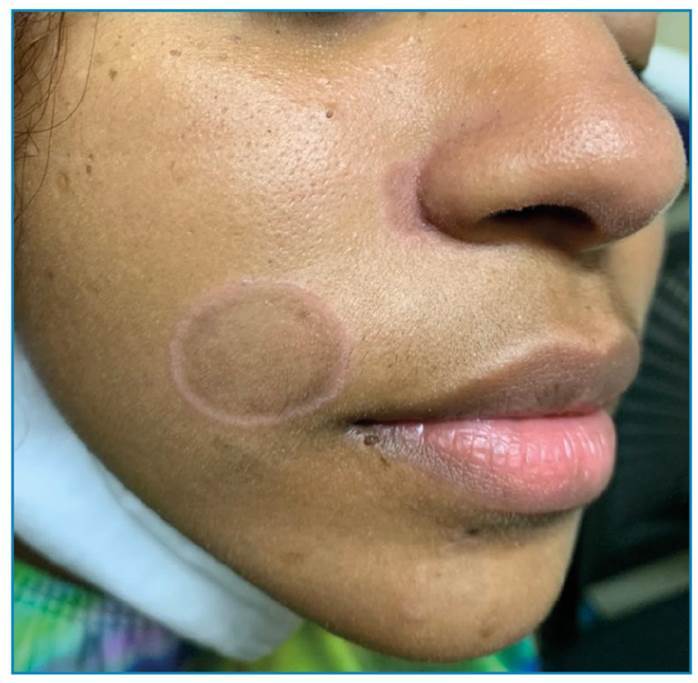
Rounded brownish plaques with erythematous edges on the face.

**FIGURE 2:**
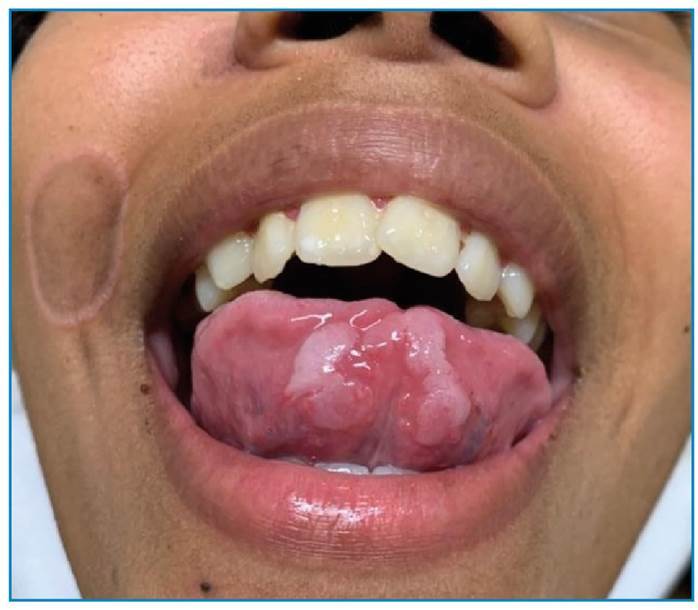
Whitish and confluent plaques on the ventral side of the tongue.

**FIGURE 3:**
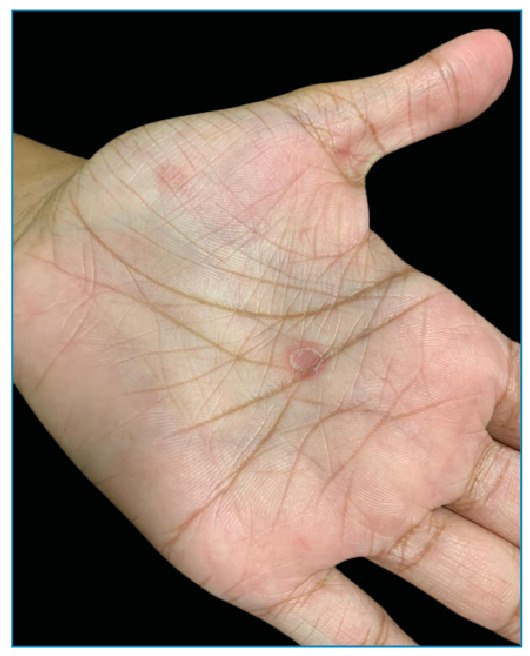
Erythematous macules with Biett collarette in palmar region.

A 15-year-old female, born in Rio de Janeiro reported the appearance of discreetly pruritic lesions on the face and left palmar region 3 months ago with no other associated symptoms. She reported having had unprotected sex in the last 3 months. Physical examination revealed two well-defined, rounded, non-scaly, brownish plaques with slightly elevated erythematous edges: one located superior to the right oral angle and the other lateral to the right nasal ala. Multiple whitish confluent plaques were observed in the ventral region of the tongue. In the left palmar region, two circular erythematous macules with peripheral whitish scaly collarettes were observed. Dermatological findings included elegant syphilides on the face (Figure 1), mucous plaques on the tongue (Figure 2), and syphilitic roseola with a Biett collarette in the palmar region (Figure 3). The clinical diagnosis was secondary syphilis, confirmed using quantitative VDRL (1/32) and rapid treponemal test (FTA-ABS) reagents. We administered 2,400,000 IU intramuscular single dose of benzathine penicillin that led to resolution of the lesions and treatment of sexual contacts^1^. Secondary syphilis manifests as various types of rashes on the skin and mucous membranes^2^. In afro-descent patients, the lesion on the face can present as elegant syphilide. It occurs infrequently around the nose and mouth, has an annular or circinate configuration, and is a characteristic finding during the secondary phase of the disease that requires further investigation for diagnostic confirmation^3^.
